# Impact of hypertensive disorders on disease progression in pregnancies affected by early‐onset fetal growth restriction

**DOI:** 10.1111/aogs.70049

**Published:** 2025-09-01

**Authors:** Basia Chmielewska, Claire Pegorie, Michelle Jie, Nishita Mehta, Daniel McStay, Amar Bhide, Basky Thilaganathan

**Affiliations:** ^1^ Fetal Medicine Unit St George's University Hospitals NHS Foundation Trust London UK; ^2^ Department of Obstetrics and Gynaecology Epsom and St Helier Hospital University Hospitals NHS Trust London UK; ^3^ Department of Obstetrics and Gynaecology Croydon University Hospital NHS Trust Croydon UK; ^4^ Vascular Biology Research Centre Molecular and Clinical Sciences Research Institute, City St George's University of London London UK

**Keywords:** chronic hypertension, hypertensive disorders of pregnancy, placental insufficiency, preeclampsia, TRUFFLE

## Abstract

**Introduction:**

Fetal growth restriction is a leading cause of perinatal morbidity, often linked to placental insufficiency. Hypertensive disorders frequently coexist with fetal growth restriction and may alter its clinical course. The objective of this study is to examine how hypertensive disorders influence the onset, progression, and timing of birth in pregnancies affected by fetal growth restriction. Secondary outcomes were indications for delivery and neonatal outcomes.

**Material and Methods:**

A retrospective cohort study of pregnancies diagnosed with fetal growth restriction prior to 36 weeks' gestation and monitored under the TRUFFLE protocol between January 2013 and July 2024 at a tertiary fetal medicine unit in the UK. Pregnancies were stratified by maternal blood pressure status: normotensive, hypertensive disorder of pregnancy, or preexisting chronic hypertension. Clinical characteristics, antenatal surveillance findings, delivery indications, and neonatal outcomes were compared between groups.

**Results:**

One hundred and ninety‐six singleton pregnancies met the inclusion criteria. 68% of the cohort were affected by chronic hypertension or new‐onset hypertensive disorders of pregnancy. Hypertensive pregnancies had significantly shorter intervals from fetal growth restriction diagnosis to delivery (9 days (IQR 5–19) for chronic hypertension, 12 days (IQR 3–24) for hypertensive disorders of pregnancy, 23 days (IQR 8–35) in normotensive pregnancies (*p* = 0.001)) and earlier gestational age at delivery (29 + 5 weeks (IQR 27 + 3–32 + 3) for chronic hypertension and 30 + 5 weeks (IQR 28 + 4–32 + 6) for hypertensive disorders of pregnancy — versus 32 + 0 weeks (IQR 29 + 1–33 + 6) in normotensive cases; *p* = 0.023). A higher proportion of hypertensive pregnancies were delivered for maternal indications (37.5% hypertensive disorders of pregnancy, 39.5% chronic hypertension) compared to 14.5% in normotensive pregnancies (*p* = 0.004), while normotensive pregnancies were more frequently delivered due to abnormal umbilical artery Dopplers (29.0% vs. 14.6% hypertensive disorders of pregnancy, 13.2% chronic hypertension; *p* = 0.041). Neonates of mothers with chronic hypertension had higher birthweight centiles (*p* = 0.004), but neonatal outcomes were comparable across all groups.

**Conclusions:**

Incidence of hypertension in the context of fetal growth restriction significantly impacts timing and gestational age of delivery and birthweight centile. An integrated approach to combine maternal and fetal monitoring in these pregnancies is required to optimize birth outcomes.

## Graphical abstract

Hypertensive disorders in fetal growth restriction halve the time to delivery and shift indications for birth, supporting integrated maternal–fetal monitoring.

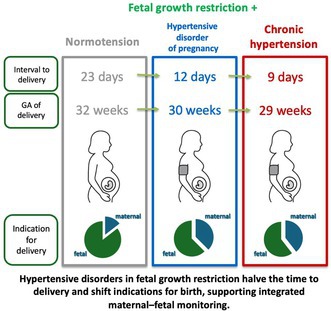

**Abbreviations**
AC: abdominal circumference
BP: blood pressure
BW: birth weight
cHTN: chronic hypertension
DV: ductus venosus
EFW: estimated fetal weight
FGR: fetal growth restriction
HDP: hypertensive disorders of pregnancy
PE: preeclampsia
UA: umbilical artery

**Key message**

Hypertensive disorders significantly shorten the interval from fetal growth restriction diagnosis to delivery and alter delivery indications, highlighting the need for integrated maternal–fetal monitoring to guide optimal timing of birth in growth‐restricted pregnancies.

## 1 INTRODUCTION

Fetal growth restriction (FGR), in which the fetus fails to achieve its biological growth potential, is associated with stillbirth and iatrogenic preterm birth, posing substantial risks of neonatal mortality, significant morbidity, and long‐term health complications.^1^, ^2^, ^3^ These risks are closely linked to both the gestational age at birth and the severity of the growth restriction.^4^ Although FGR can be caused by a variety of factors such as congenital infection and genetic abnormalities, the predominant etiology of FGR is poor placental function as a consequence of uteroplacental dysfunction.^5^, ^6^ Maternal hypertension, either preexisting or as preeclampsia (PE) developing in the second half of pregnancy, often coexists in pregnancies affected by FGR.^5^, ^6^ PE is also a consequence of uteroplacental dysfunction and women with PE have a higher incidence of FGR, preterm birth, and stillbirth.^7^ Pregnancies affected by both fetal growth restriction and preeclampsia have considerably poorer maternal and neonatal outcomes.^6^

Since the publication of results from the TRUFFLE trial, which showed improved survival without neurological impairment, early‐onset FGR before 34 weeks' gestation is widely managed using clinical guidelines that recommend surveillance of umbilical artery (UA) and ductus venosus (DV) Doppler indices and computerized cardiotocography (cCTG).^8^ The frequency of surveillance is dependent on the severity of FGR and UA abnormalities, with elective birth recommended if critical changes are noted in DV, UA, and cCTG. Although some guidelines recommend a blood pressure (BP) assessment at FGR diagnosis, the practice of more comprehensive BP surveillance of women affected by FGR pregnancies is paradoxically not well established, despite the established recommendation for regular BP monitoring in women with uncomplicated pregnancies.^9^, ^10^ Cohort outcomes from the TRUFFLE trial showed that over two thirds of women with growth‐restricted fetuses had a hypertensive condition at delivery, of which a quarter were delivered solely on maternal indication.^11^ There is a paucity of information about the impact of the development of PE on disease progression and outcome in early‐onset FGR.

The aim of this study is to investigate how hypertensive disorders affect the onset and progression of fetal growth restriction and explore the temporal nature of the clinical manifestations of uteroplacental dysfunction.

## 2 MATERIAL AND METHODS

### 2.1 Study design

This was a retrospective cohort study of all singleton pregnancies affected by fetal growth restriction delivered prior to 36 weeks' gestation between January 2013 and July 2024, booked at or referred to St George's University Hospitals NHS Foundation Trust. Pregnancies affected by fetal hydrops, premature prolonged rupture of membranes (PPROM) prior to FGR diagnosis, major fetal abnormalities (chromosomal abnormalities, fetal or placental chromosomal mosaicism, abnormalities of any major fetal organs) or FGR secondary to independent placental pathologies, including chorioangiomas and chronic histiocytic intervillositis (CHI), were excluded from the cohort. CHI cases had a history of recurrent fetal loss and were excluded because fetal demise happens in the absence of Doppler abnormalities and therefore are not suitable for TRUFFLE monitoring. Pregnancies that ended before commencement of monitoring on the TRUFFLE protocol were also excluded from analysis.

### 2.2 Study cohort

The cohort was identified from the ultrasound database and maternity birth registry. Diagnosis of FGR was according to the inclusion criteria for the TRUFFLE study and defined as an estimated fetal weight (EFW) or abdominal circumference (AC) below the 10th centile, combined with a raised umbilical artery pulsatility index (PI) of greater than the 95th centile.^8^ The cohort was divided into three groups: normotensive, those developing hypertensive disorders of pregnancy (HDP) and having pre‐pregnancy chronic hypertension (cHTN). Chronic hypertension was defined as medicated hypertension prior to pregnancy or before 20 weeks' gestation. Hypertensive disorder of pregnancy was defined as blood pressure readings above or equal to, that is, systolic BP ≥140 mmHg and/or diastolic BP ≥90 mmHg, in repeated measurements after 20 weeks of gestation, as defined by International Society for the Study of Hypertension in Pregnancy (ISSHP) guidelines.^12^

### 2.3 Outcome measures

Sociodemographic characteristics and maternal factors were included in the analysis. Gestational age was determined from crown‐rump length measurement in the first trimester. Umbilical artery PI centile, EFW centile, and birthweight (BW) centile were calculated using the Intergrowth‐21 formulae.^5^ Neonatal death was defined as death within 28 days of birth. Indication for delivery was categorized into 4 groups: early or late DV changes (PI >95th centile or absent A‐wave), CTG concerns (low short‐term variation or decelerations), maternal reasons, and absent or reversed end‐diastolic flow in the umbilical artery as per TRUFFLE protocol and safety net criteria.^8^ Maternal reasons included severe preeclampsia, Hemolysis‐Elevated Liver Enzymes and Low Platelets (HELLP) syndrome, the presence of multiple interacting maternal comorbidities, and circumstances in which the multidisciplinary team determined, based on the comprehensive clinical context, that delivery was indicated.

### 2.4 Statistical analyses

Statistical analysis was performed using IBM*SPSS*Statistics version 30. Figures were produced using GraphPad Prism version 10. Categorical variables are presented as frequencies and percentages, and continuous variables as medians and interquartile ranges (IQR). Bivariate analysis was performed using the χ2 test (or Fisher exact test as appropriate) for categorical variables and ANOVA for continuous variables. All reported p‐values are two‐sided, with values of less than 0.05 indicating statistical significance.

## 3 RESULTS

**FIGURE 1:**
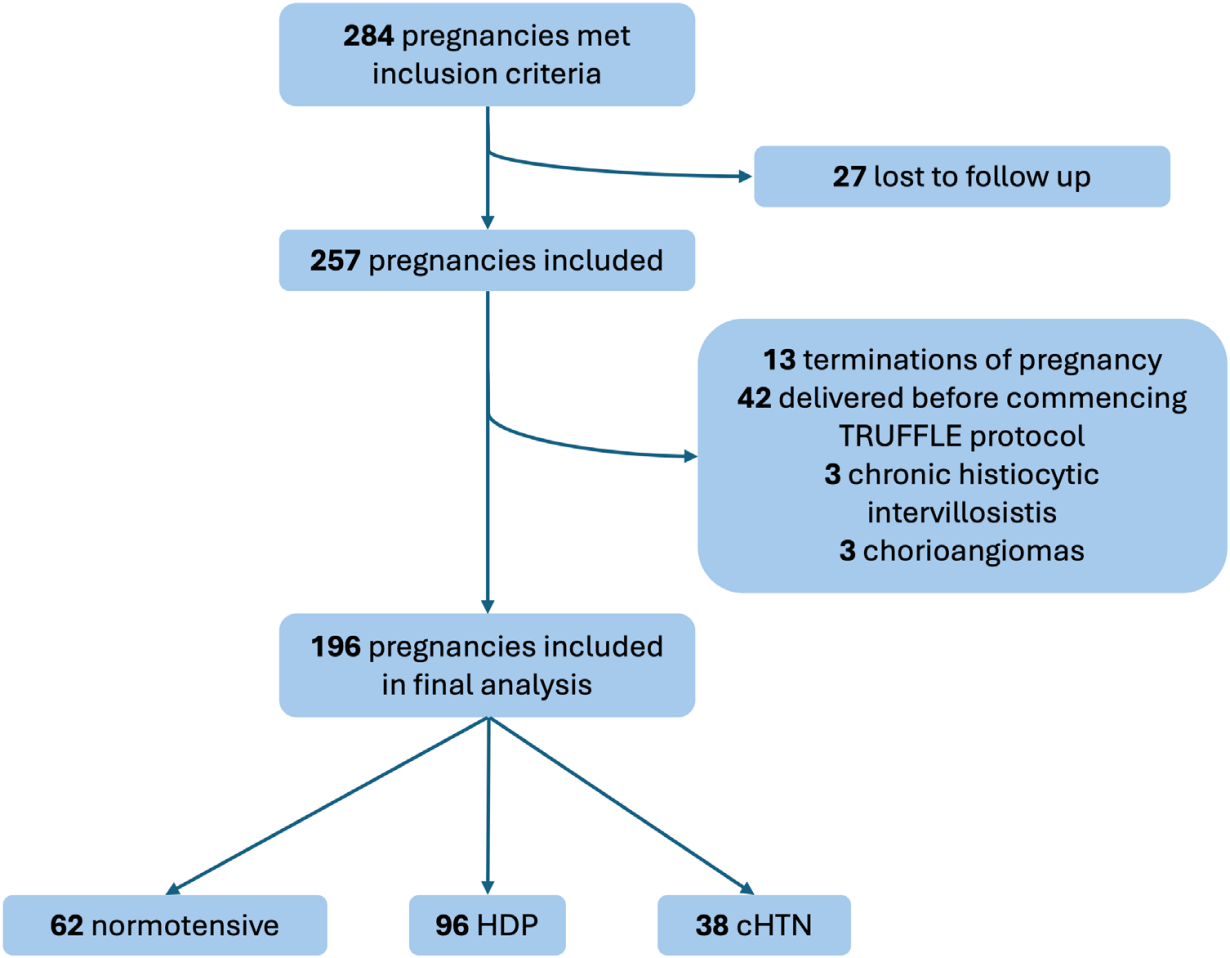
Flowchart showing identification of study cohort. HDP = hypertensive disorders of pregnancy. CHTN = chronic hypertension.

The study cohort included 196 of the 284 FGR pregnancies identified from the ultrasound and delivery databases (Figure 1). Exclusions were made for birth before commencing the TRUFFLE protocol (*n* = 42), medical termination (*n* = 13), FGR from other pathologies (*n* = 6) and loss to follow‐up (*n* = 27). The cohort was divided into 3 groups: normotensive (*n* = 62), with HDP (*n* = 96) and pre‐pregnancy cHTN (*n* = 38). Demographic data are shown in Table 1 with only the median maternal age differing significantly between the normotensive, HDP, and cHTN subgroups (*p* = 0.004). The median gestational age of diagnosis of FGR was 28 + 1 weeks (26 + 1–30 + 2 IQR) with 171 (87.2%) diagnosed with FGR prior to 32 weeks' gestation (Table 2).

**TABLE 1:** Demographic data of pregnancies affected by early‐onset fetal growth restriction.

	All (*n* = 196)	Normotensive (*n* = 62)	HDP (*n* = 96)	cHTN (*n* = 38)	*p*‐value
Maternal age (years)	32.0 (27.0–36.8)	31.0 (27.0–35.0)	32.0 (26.0–35.0)	36.0 (29.0–39.0)	0.004
BMI (kg/m^2^)	28.0 (24.0–32.0)	28.5 (23.0–34.0)	27.0 (24.0–31.0)	29.5 (25.0–32.0)	0.299
Para 0	109 (55.6%)	35 (56.5%)	55 (57.3%)	19 (50%)	0.165
Para 1	52 (47.7%)	20 (32.3%)	19 (19.8%)	13 (34.2%)	
Para ≥2	35 (17.9%)	7 (11.2%)	22 (22.9%)	6 (15.8%)	
Smoking	18 (9.2%)	7 (11.3%)	8 (8.3%)	3 (7.9%)	0.783

*Note*: Cohort divided into three groups: those not affected by hypertension (nHTN), those with hypertensive disorders of pregnancy (HDP) and those with chronic hypertension (cHTN). Data displayed as *n* (%) or median (IQR).

**TABLE 2:** Antenatal data, delivery data, and neonatal outcome data of pregnancies affected by fetal growth restriction.

	All (*n* = 196)	Normotensive (*n* = 62)	HDP (*n* = 96)	cHTN (*n* = 38)	p‐value
Antenatal data					
GA of FGR diagnosis (weeks)	28 + 1 (26 + 1–30 + 2)	27 + 5 (24 + 4–30 + 6)	28 + 1 (26 + 5–30 + 1)	28 + 0 (25 + 5–29 + 6)	0.515
Diagnosis to delivery interval (days)	14 (5–29)	23 (8–35)	12 (3–24)	9 (5–19)	0.001
Last cCTG STV (mS)	5.9 (4.3–7.1)	5.7 (4.1–8.1)	6.1 (4.1–7.1)	5.8 (4.5–6.7)	0.616
Last UmbA PI	2.12 (1.65–2.90)	2.17 (1.65–3.15)	2.07 (1.64–2.74)	2.23 (1.69–2.83)	0.405
Last DV PI	0.76 (0.60–1.04)	0.79 (0.59–1.19)	0.75 (0.63–0.96)	0.77 (0.58–1.01)	0.532
Neonatal deaths	10 (5.1%)	5 (8.0%)	5 (5.2%)	0 (0.0%)	0.198
Delivery data					
GA at delivery (weeks)	30 + 6 (28 + 3–33 + 3)	32 + 0 (29 + 1–33 + 6)	30 + 5 (28 + 4–32 + 6)	29 + 5 (27 + 3–32 + 3)	0.023
Birth weight (g)	960 (660–1345)	1080 (601–1462)	960 (680–1285)	840 (675–1500)	0.678
Birth weight centile	2.06 (0.14–5.93)	0.67 (0.03–4.14)	2.06 (0.20–5.52)	4.90 (1.38–14.30)	0.004
Delivered for DV Doppler	37 (18.9%)	15 (24.2%)	16 (16.7%)	6 (15.8%)	0.405
Delivered for cCTG (STV or decelerations)	57 (29.1%)	18 (29.0%)	27 (28.1%)	12 (31.6%)	0.990
Delivered for maternal reasons	60 (30.6%)	9 (14.5%)	36 (37.5%)	15 (39.5%)	0.004
Delivered for UmbA Doppler	37 (18.9%)	18 (29.0%)	14 (14.6%)	5 (13.2%)	0.041
Birth outcomes					
Abruption	3 (1.5%)	0 (0.0%)	3 (3.1%)	0 (0.0%)	0.301
Livebirths	191 (97.4%)	60 (96.8%)	93 (96.9%)	38 (100%)	0.710
Stillbirths	5 (2.6%)	2 (3.2%)	3 (3.1%)	0 (0.0%)	
Neonatal deaths	10 (5.1%)	5 (8.0%)	5 (5.2%)	0 (0.0%)	0.198
Neonatal outcomes					
1‐min APGAR	7 (4–7)	7 (4–9)	8 (5–9)	7 (4–9)	0.616
5‐min APGAR	9 (8–10)	9 (8–10)	9 (7–10)	9 (7–9)	0.697
HIE	1 (0.5%)	0 (0.0%)	1 (1.0%)	0 (0.0%)	1.000
Required ventilation	80 (40.8%)	23 (37.1%)	40 (41.7%)	17 (44.7%)	0.784
Ventilation (days)	5 (2–20)	4 (2–22)	5 (1–17)	6 (1–30)	0.281
Admission to NNU	185 (96.8%)	58 (96.7%)	89 (95.7%)	38 (100%)	0.490

*Note*: Cohort divided into three groups: normotensive, those with hypertensive disorders of pregnancy (HDP) and those with chronic hypertension (cHTN). Data displayed as *n* (%) or median (IQR).

Abbreviations: cCTG, computerized cardiotocography; DV, ductus venosus; GA, gestational age; STV, short‐term variation; UmbA PI, umbilical artery pulsatility index.

**FIGURE 2:**
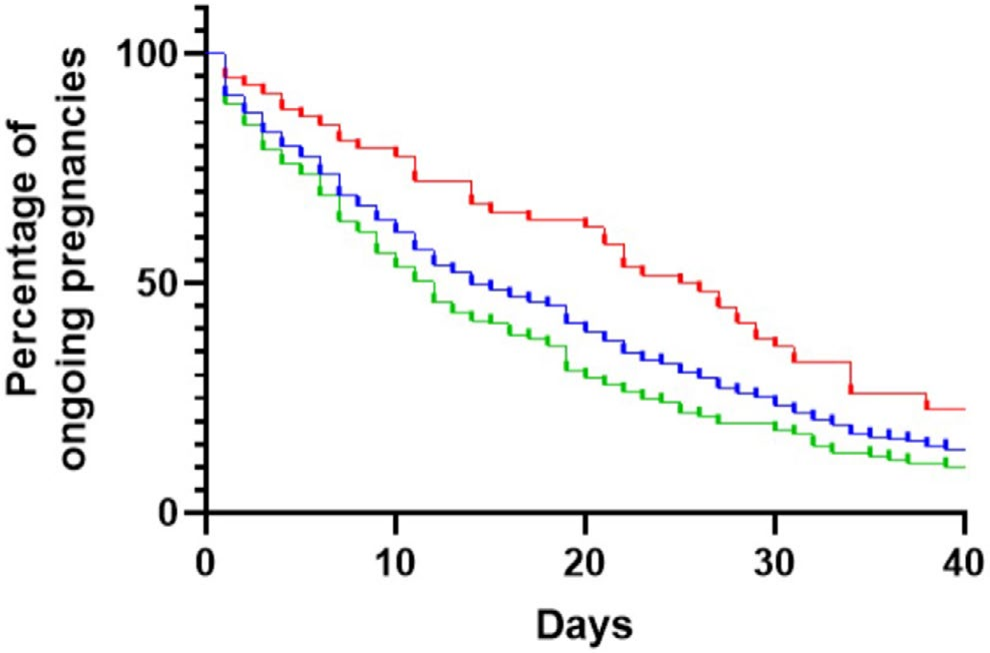
Survival curve showing the interval between diagnosis of FGR to delivery in the entire cohort (blue line). There was a significant difference (*p* = 0.0019) in the diagnosis to birth interval for FGR pregnancies with (green line) and without (red line) hypertension.

**FIGURE 3:**
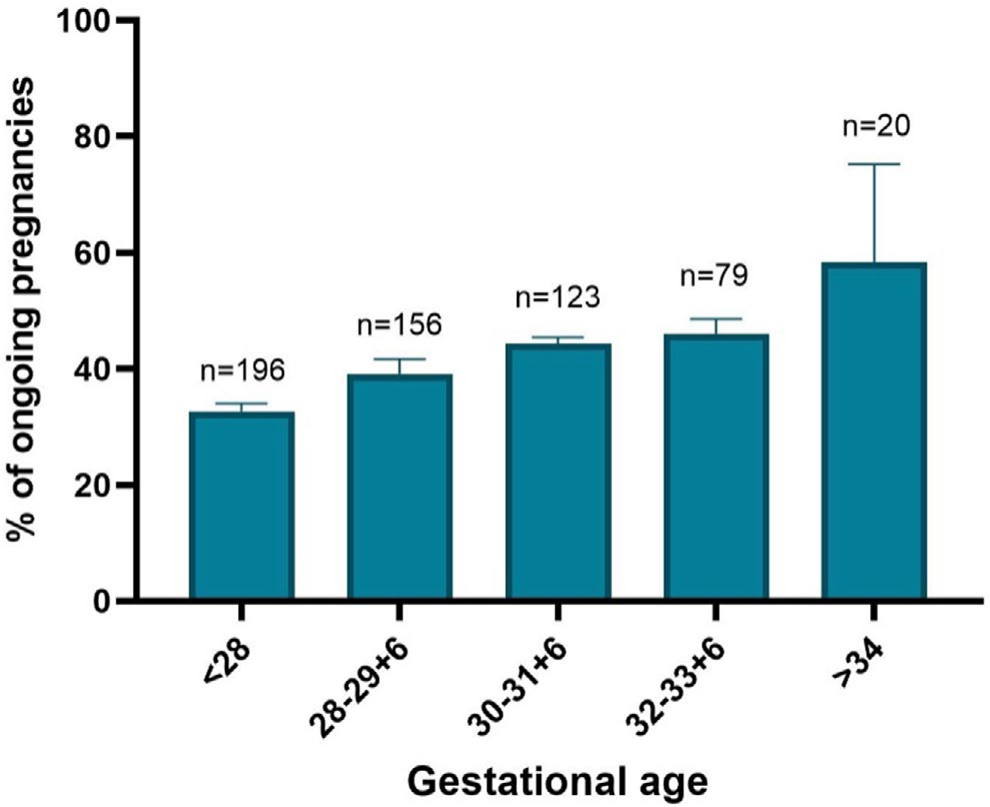
Percentage of ongoing FGR pregnancies affected by hypertension according to gestational age (mean percentage and standard deviation).

The median interval from FGR diagnosis to delivery varied significantly (*p* = 0.001) from 9 days (IQR 5–19) in the cHTN, 12 days (IQR 3–24) in the HDP and 23 days (IQR 8–35) in the normotensive groups (Figure 2). Consistent with this finding, the gestational age at birth increased significantly (*p* = 0.023) from 29 + 5 weeks (27 + 3–32 + 3 IQR) in cHTN through to 32 + 0 (29 + 1–33 + 6 IQR) in normotensive women (p = 0.023). The proportion of ongoing pregnancies affected by hypertension (HDP and cHTN) also increased with gestational age (Figure 3). The indication for elective birth was different across groups, with a higher proportion delivered for maternal reasons in the HDP and cHTN groups (39.4% and 39.5%, respectively) compared with the normotensive group (15.0%, *p* = 0.003). A higher proportion of the normotensive group were delivered for abnormal umbilical artery (30.0%) compared to the HDP (17.0%) and cHTN (13.2%, *p* = 0.039) groups. A third of the cohort was delivered due to CTG abnormalities (19.4% for reduced STV, 9.7% for repetitive decelerations), and this was consistent across all groups (Table 2).

There were 97.4% of births that were live births and 2.6% that were stillbirths, with no difference between the three groups (*p* = 0.710). The median birthweight centile was significantly higher (*p* = 0.004) in cHTN (4.9; IQR 1.38–14.30) than in the HDP (2.06; IQR 0.20–5.52) and normotensive groups (0.67; IQR 0.03–4.14 IQR). There were no differences in neonatal outcomes between groups with median APGAR scores of 7 (IQR 4–7) at 1 min and 9 (IQR 8–10) at 5 min. One baby was diagnosed with hypoxic ischemic encephalopathy (HIE) from the HDP group. Of the 185 (96.8%) babies admitted to the neonatal unit, 80 (40.8%) required ventilatory support.

## 4 DISCUSSION

The findings of this study of early‐onset FGR pregnancies are that about 50% of pregnancies will develop new‐onset HDP while being monitored with the TRUFFLE protocol, a similar proportion to those demonstrated in the STRIDER and TRUFFLE studies.^11^, ^13^ The impact of both HDP and preexisting hypertension is to halve the FGR diagnosis to delivery interval compared to normotensive pregnancies. Despite earlier birth for maternal reasons in HDP and cHTN pregnancies, the neonatal outcomes in both normotensive and hypertensive women were equally good with >95% intact survival of infants.

This study includes data collected over an 11‐year period from a large multi‐ethnic population of women covering a wide geographical area with comprehensive antenatal and neonatal data. The main limitations of this study are that confounding factors that may not have been fully accounted for, such as other maternal comorbidities or fetal variables. These factors could have influenced both fetal growth and hypertension but were not controlled for in this analysis. Additionally, the observational nature makes it difficult to determine causality, but this was not a primary endpoint of the study.

Over two thirds of FGR pregnancies will be affected by hypertension by the time of birth. Crucially, as gestational age advances, the proportion of pregnancies complicated by maternal hypertension increases (Figure 3). Those with either preexisting hypertension or new‐onset HDP will have a shorter interval between diagnosis of FGR to delivery and will deliver at an earlier gestation compared to normotensive pregnancies. Over a third of those women will be delivered for a maternal indication, of which the predominant reason is severe PE or deteriorating blood pressure control. Maternal hypertension reflects acceleration in the pathophysiological processes underlying placental insufficiency, which requires earlier intervention (Figure 2). The new onset of hypertension in pregnancy should trigger an increase in fetal monitoring frequency, as it signals the acute deterioration of uteroplacental insufficiency.

Paradoxically, birthweight centiles were higher in hypertensive pregnancies despite an earlier gestational age of delivery. The most likely reason for this finding is that earlier birth for HDP results in an interruption of the progressive deterioration in fetal growth with prolonging gestation. A similar finding was seen in the DIGITAT randomized control trial of term small‐for‐gestational age pregnancies at term, which demonstrated that conservatively managed pregnancies were born at significantly lower birthweight centiles than those induced at the time of diagnosis.^14^ It is widely accepted that FGR and PE are closely associated, and this data suggests that they may be sequential manifestations of placental insufficiency. Our findings advocate for an increased frequency of monitoring of maternal blood pressure and proteinuria in cases of fetal growth restriction.

Our results support the hypothesis that FGR and HDP should be considered as manifestations of the same underlying disease process, rather than separate disease entities or conditions themselves. Both outcomes arise because of a common etiology from placental dysfunction, but with different pathophysiological mechanisms resulting in these well‐recognized maternal and fetal outcomes. Recognizing FGR and HDP as part of a spectrum of disease for placental dysfunction has important clinical and research implications. For example, screening tests typically feature an outcome of FGR, HDP, or stillbirth in isolation rather than as outcomes of the same disease, and as such, are destined to underperform. Similarly, monitoring and treatment regimens should consider both outcomes as inevitable consequences of placental dysfunction.

## 5 CONCLUSION

The findings of this study highlight the importance of closely monitoring blood pressure in pregnancies complicated by early‐onset FGR, as this significantly impacts the timing of birth and birthweight centile. An integrated approach to combine maternal and fetal monitoring in these pregnancies is required to optimize birth outcome. Furthermore, the strength and temporal nature of the relationship between FGR and HDP support the hypothesis of uteroplacental dysfunction being the common etiology for these pregnancy complications.

## AUTHOR CONTRIBUTIONS

BC and CP: planning, data collection and analysis, write up. MJ: data collection and analysis, write up. NM and DM: data collection. AB: supervision of data analysis and write up. BT: concept, planning, supervision of data collection, analysis and write up.

## FUNDING INFORMATION

No specific funding was received for this study.

## CONFLICT OF INTEREST STATEMENT

No conflict of interest to declare by any of the authors.

## ETHICS STATEMENT

The present study was deemed not to require ethics approval or signed patient consent, in accordance with the UK Health Research Authority decision tool.

## Data Availability

The data that support the findings of this study are available from the corresponding author upon reasonable request.
